# Attentional Avoidance for Guilty Knowledge Among Deceptive Individuals

**DOI:** 10.3389/fpsyt.2019.00114

**Published:** 2019-03-12

**Authors:** Kiho Kim, Go-eun Kim, Jang-Han Lee

**Affiliations:** Clinical Neuro-Psychology Laboratory, Department of Psychology, Chung-Ang University, Seoul, South Korea

**Keywords:** attentional bias, attentional avoidance, deception detection, guilty knowledge test, concealed information test, eye-movement

## Abstract

The purpose of the present study is to differentiate between innocent suspects who have knowledge of crime information and guilty suspects. The study investigated eye-movement differences among three groups: a guilty group who took part in a mock crime, an innocent-aware group who did not commit a mock crime but were exposed to the crime stimuli, and an innocent-unaware group who neither committed a mock crime nor had crime-relevant information. Each group's eye movements were tracked while all participants viewed stimuli (crime-relevant, crime-irrelevant, and neutral). The results revealed that the guilty group not only viewed all stimuli later than the other groups, they also viewed crime-relevant and crime-irrelevant stimuli for a shorter time period than the innocent-aware group; the innocent-aware group focused their attention on crime-relevant and crime-irrelevant stimuli longer than neutral stimuli, and the innocent-unaware group showed no differences in their attention focus among all types of stimuli. This present study suggests that guilty individuals show attentional avoidance from all stimuli in a lie detection situation, whereas innocent-aware and innocent-unaware individuals did not show avoidance responses.

## Introduction

The Guilty Knowledge Test (GKT) or the Concealed Information Test (CIT) is a deception detection method and is intended to establish the existence of a specific memory trace (1–3). The GKT is based on the assumption that suspects who possess knowledge about specific crime related details will be physiologically more reactive to crime-relevant questions than crime-irrelevant questions, by utilizing a series of multiple-choice questions, each having one crime-relevant question and several control questions (4). GKT relies on a solid scientific principle, called an orienting response (OR), which is an elicited response caused by a novel stimulus or a familiar stimulus with relevance or “signal value” (5, 6), and it has been shown that guilty knowledge has an added signal value (7). That is, people who have guilty knowledge show a stronger OR to crime-relevant questions than to other questions, whereas all questions elicit equivalent responses from truth tellers. Laboratory research has reported that the GKT has high validity coefficients for the differentiation between guilty and innocent persons on the basis of autonomic measures such as skin conductance responses, respiration, heart rate, the P300 event-related potential (8–11), and can be generalized to the criminal field (12, 13).

However, the GKT also has the possibility of false positive errors because it may not work correctly when innocent suspects are exposed to crime-related information (14–17). It is not easy to keep salient features of a crime from being leaked to the innocent group, and the leakage of the critical features of the crime might put the innocent group in substantial danger because knowledge of the critical crime stimuli might be sufficient for producing differential responses to the stimuli. Therefore, it is widely known that false positive errors, where the innocent group is judged as guilty, can be controlled as long as information about the crime is not leaked to innocent group in the GKT.

Because the GKT may not work with an innocent group that has guilty knowledge, Ben-Shakhar et al. (18) attempted to identify the effects of awareness of crime-relevant information on the deception detection with the GKT. They investigated whether introducing target stimuli, to reduce false positive outcomes, occurred from the leakage of crime-relevant information to the innocent group. In their study, they introduced target items to which participants must respond while answering the GKT questions with the purpose of drawing the attention of informed innocent suspects. As a result, the informed innocent group showed relatively larger electrodermal responses to the critical stimuli than uninformed ones—but not as large as the responses of the guilty group. However, it is a hasty conclusion to suggest that the informed innocent group attended to target stimuli at a level near that of the guilty group, because the study did not directly measure the effect of the target items in drawing attention. Therefore, it remains unclear whether discrimination between informed innocent and guilty suspects is possible (19, 20).

It is known that not only physiological activity, but also attentional processes are involved in responses to guilty knowledge, as a component of the OR (21). Indeed, several authors have argued that the main function of the OR is to enhance information processing, which is achieved by not only directing the senses to the stimulus but also allocating attention toward it. Both novel and significant stimuli are associated with an allocation of attention, as measured by task interference on a concurrent reaction time task (22, 23). In addition, Verschuere et al. (24) found that guilty knowledge elicits a signal-OR and therefore demands attentional resources with a probe classification task. Therefore, it is reasonable to think that guilty knowledge demands attention. However, there has been no indication of spatial shifting of attention on guilty knowledge thus far, and it remains unclear whether participants would shift attention either toward or away from guilty knowledge. An eye-tracking technique can be an effective way to investigate the direction of attention because eye tracking is a continuous method of measuring eye movement, which allows for the direct observation of attentional engagement, shift, and a disengagement pattern (25). The eye-tracking device not only provides a highly direct measure of visual attention but also allows continuous measurement of gaze patterns.

Gaze patterns reveal complex information processing that can be explained as an attentional bias that involves both autonomic and controlled processes (26, 27). Eye tracking literature defines initial gaze fixation or first fixation latency “where one looks” as OR /initial orienting of overt attention to a stimulus (often a more automatic process); and dwell time or fixation time as the later process of “how long one looks,” a rather strategically controlled process (28, 29). That is, it is likely that people who have guilty knowledge initially fixate their eyes toward crime-relevant information automatically because of the OR but subsequently show cognitive eye movements as a manifestation of strategic behavior in controlled processes. Recently, Kim et al. (30) attempted to identify whether or not liars, as compared to truth tellers, would have an attentional bias for guilty knowledge using the eye tracker. As a result, both the guilty and the innocent groups initially fixated on crime-relevant stimuli rather than on both crime-irrelevant and neutral stimuli. In addition, the guilty group showed a longer dwell time for neutral stimuli than the innocent group did, although there was no difference between the two groups for crime-relevant and irrelevant stimuli. These findings possibly indicate that the guilty group reflexively moved their eyes toward crime-relevant stimuli as an OR, but they strategically diverted their attention from these stimuli so as not to be found guilty of theft. It has been found that liars use an “avoid and escape” strategy when confronted with deceptive evidence during communication (31). It might be assumed that guilty people who have guilty knowledge show differential responding to crime-relevant information than innocent people who have guilty knowledge and innocent people who have no guilty knowledge. Therefore, there is a need to investigate in order to differentiate innocent suspects who have knowledge of crime information from guilty suspects, using attentional bias regarding crime information, by measuring eye movement.

The purpose of this study was to investigate attentional bias regarding crime information, by measuring eye movement and to reveal differences in attentional patterns between guilty and innocent-aware groups. We investigated the eye-movement differences among three groups: a guilty group who committed a mock crime, an innocent-aware group who did not commit a mock crime but were naturally exposed to guilty knowledge, and an innocent-unaware group who did not have any knowledge of the crime and did not commit a mock crime. We predicted that the guilty group would show a shorter first fixation time and a shorter dwell time toward crime-relevant stimuli than crime-irrelevant and neutral stimuli. In addition, we expected that the innocent-aware group would show a shorter first fixation time and a longer dwell time toward both crime-relevant and crime-irrelevant stimuli than neutral stimuli. Finally, we expected that there would be no differences in a first fixation time and a dwell time to all types of stimuli in the innocent-unaware group.

## Material and Methods

### Participants

A total of 60 undergraduate students from Seoul, Korea were recruited for this experiment. All participants were physically and psychologically healthy, and their state of health was checked by an interview. Participants were randomly assigned to one of the three groups: a guilty group who committed a mock crime and possessed crime-relevant knowledge, an innocent-aware group who possessed crime-relevant information even though they did not take part in the mock crime, and an innocent-unaware group who did not have any knowledge of the mock crime. Of all participants, three from the guilty group, five from the innocent-aware group, and one from the innocent-unaware group were removed as outliers—three because their dwell time results were more than 2 SD from the mean (three had unusually variable dwell time), five because their first fixation time results were more than 2 SD from the mean (five had unusually variable first fixation time) and one had almost half of the data missing due to measurement errors. Finally, the guilty group consisted of 17 participants (six males, mean age = 22.56; *SD* = 2.10), the innocent-aware group consisted of 15 participants (nine males, mean age = 23.67, *SD* = 2.82), and the innocent-unaware group consisted of 19 participants (nine males, mean age = 21.45, *SD* = 2.16).

### Apparatus and Materials

Eye movements for all participants were recorded with an eye-tracking device (iView XTM Red—IV Eye Tracking System, Sensomotoric Instruments GmbH, Berlin, Germany) at a sampling rate of 60 Hz. In order to analyze the eye-movement data, we used the Begaze (SMI, Berlin, Germany) software package, which provided a variety of gaze information, such as how long they fixated their attention, where they focused, how many times they saw the specific location or stimulus, and so on. Each participant was seated at a desk, at a distance of 70 cm from a 23-inch wide monitor (1,920 × 1,080), and the eye tracker allowed the participants to naturally move their heads and eyes without any attached sensors. The eye movements that were stable for at least 80 ms within the visual angle of 1.4° were defined as a fixation (28).

Three types of stimuli were used: crime-relevant, crime-irrelevant, and neutral stimuli. Crime-relevant stimuli comprised of four items that were used in the mock crime: black USB, white envelope, purple legal seal, and black pen. Crime-irrelevant stimuli comprised of four items that were similar to the crime-relevant stimuli in shape but were not used for the mock crime: silver USB, purple postcard, unofficial seal, and pencil. Finally, neutral stimuli comprised of four items that were not exposed to participants during the experiment: Thermos, stapler, toothbrush, and felt-tipped pen. Twenty-four people other than the experimental participants rated the valence and arousal of each stimulus with a 7-point Likert scale, with 1 labeled as “very unpleasant” and “calm,” and 7 labeled as “very pleasant” and “arousing.” There were no differences in the mean valence and arousal rating among the three stimuli types. Each picture was 95 mm high by 130 mm wide when displayed on the screen, and the distance between their inner edges was 30 mm. The distance between the two probe positions was 105 mm (visual angle of 5.4°). The task consisted of 36 pairs, Crime-relevant & Crime-irrelevant, Crime-relevant & Neutral, and Crime-irrelevant & Neutral which were presented on one screen at the same time. The pairs were presented in a counterbalanced order between the left and right sides of the screen. A total of 72 trials were performed in two blocks.

### Measures

#### Recognition Test

A recognition test was conducted to determine how well-participants remembered the crime-relevant and crime-irrelevant stimuli. The test consisted of 12 single-selection questions (four questions of crime-relevant stimuli, four questions of crime-irrelevant stimuli, four questions of neutral stimuli), and participants were asked to mark an X in the appropriate answer (i.e., 1: the stimuli you stole during the experiment, 2: the stimuli you did not steal during the experiment, and 3: you do not remember the stimuli or do not know the answer). Therefore, the correct answer was different for each group. One point was given if the answer was correct, if not then 0 points were given adding up to the total score of 12 points.

### Procedure

Upon their arrival, participants were given a brief description of the experiments and their rights as a research participant and signed an informed consent form. Both the experiment and the informed consent was approved by the institutional review board of Chung-Ang University. Afterward, they were informed that they would take part in an experiment on detecting deception and instructed to try not to be judged as guilty. Then, they were randomly assigned to one of the three groups: guilty, innocent-aware, and innocent-unaware. The mission for the guilty group was to enter the teaching assistant's office, steal money (~50 dollars) in a white envelope, then falsify an account book file in the black USB to cover up for stealing the money. After, they were to write out a fake receipt using a black pen, and then stamp a purple seal on the fake receipt without getting caught. The mission for the innocent-aware group was to go to the teaching assistant's office, ask someone for permission to bring eight items, including crime-relevant and crime-irrelevant stimuli, as an errand for the assistant. There was no specific mission for the innocent-unaware group. The innocent-unaware group just stayed in the laboratory for about 15 min doing nothing. After each mission was completed, all participants came back to the psychology laboratory and moved to the next room for the eye-tracking experiment. Then, we presented crime-relevant and crime-irrelevant stimuli as criminal evidence to the guilty and innocent-aware groups on the computer screen on the desk before eye tracking, whereas the innocent-unaware group did not receive such information. A total of eight stimuli were presented one by one for a 1,000 ms, and both the guilty and innocent-aware groups were informed that these stimuli were criminal evidence of a theft case in the laboratory. Therefore, the guilty group was exposed to crime-relevant knowledge, but individuals in this group knew the difference between crime-relevant and crime-irrelevant stimuli. While the innocent-aware group was exposed to crime-relevant knowledge, but individuals in this group could not differentiate between crime-relevant and crime-irrelevant stimuli. The innocent-unaware group was not exposed to crime-relevant knowledge at all. All participants were required to answer “No” when asked if they had committed a theft crime, and their eye movements were recorded while they looked at the pairs of stimuli that included crime-relevant information (free-viewing task).

**Figure 1:**
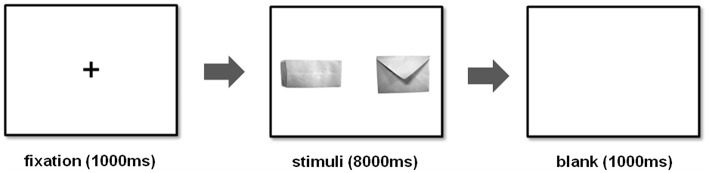
An example of the computer screen as it appeared to the subjects during the task.

Each trial started with a central cross-fixation for 1,000 ms, followed by a pair of stimulus shown side-by-side for 8,000 ms; then, a blank screen was presented for 1,000 ms for a given inter-trial interval before the start of the next trial (Figure 1). In order to control leftward or rightward bias, the location of the stimulus was counter-balanced (32). A total of 72 trials were conducted, and one stimulus was located on the left side on the screen and the other on the right side. All participants were required to maintain fixation until target stimuli appearance, and fixation behavior of the subjects was controlled prior to each trial, in that way the next trial only started if the subject fixated the cross-fixation cross for more than 300 ms. Participants' eye movements were recorded with an eye-tracker while they viewed stimuli displayed on the computer monitor. After the experiment, all participants were asked to perform a recognition test, were debriefed about the experiment and payment procedure, and were given 5,000 Won (~5 US dollars) as a reward. In addition, they were each asked not to share any information with anyone who might participate in the experiment in the future.

### Data Analysis

SPSS 15.0 for windows was used for the analyses. The changes in participants' eye movements while they were exposed to stimuli displayed on the computer monitor were measured. An area of interest (AOI) was designated to cover each picture, and the eye movements were examined in terms of fixations recorded within an AOI. In order to investigate the total amount of time spent at each stimulus (dwell time) and the amount time until the first fixation (first fixation time) in each group, a 3 (group: guilty, innocent-aware, innocent-unaware) as a between-subject factor × 3 (stimuli: crime-relevant, crime-irrelevant, neutral) as a within-subject factor repeated measures analysis of variance (ANOVA) was conducted, and degrees of freedom were adjusted with the Greenhouse-Geisser epsilon to correct for violations of the sphericity assumption.

## Results

### Sample Characteristics

There were no significant gender differences among the three groups, χ^2^(2) = 3.45, *p* = 0.18, *n.s*. In addition, there were no significant age differences among the three groups, *F*_(2,48)_ = 1.42, *p* = 0.25, *n.s*.

### Recognition Test

The number of correctly remembered items in the recognition test was 11.17 out of 12 details (*SD* = 0.59) for the guilty group, 11.80 (*SD* = 0.41) for the innocent-aware group, and 11.74 (*SD* = 0.56) for the innocent-unaware group. The one-way ANOVA on the number of correctly recognized items revealed no significant effect of the factor group, *F*_(2,48)_ = 0.13, *p* = 0.88, *n.s*., indicating that participants in all groups remembered the crime-relevant and/or crime-irrelevant stimuli well-according to each group's mission and did not differ in their recognition rates.

### Dwell Time

**Figure 2:**
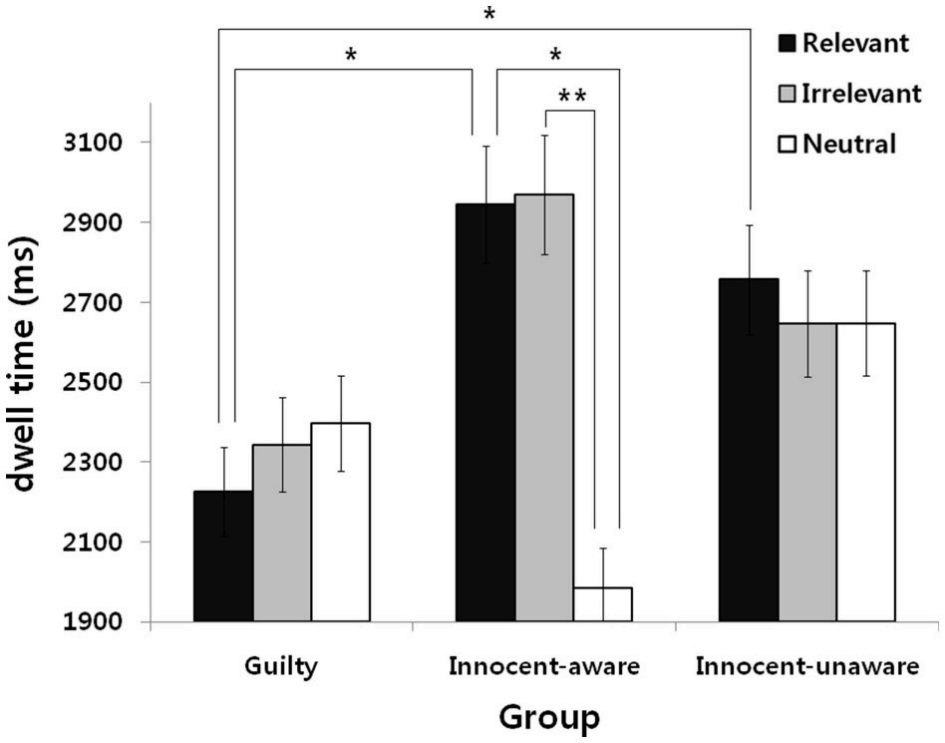
Dwell time for the three stimulus types with respect to the subject group. Means and standard error (^*^p < 0.05, ^**^p < 0.01) are shown.

Degrees of freedom were adjusted with the Greenhouse-Geisser epsilon to correct for violations of the sphericity assumption. The results revealed significant group × stimuli interaction, *F*_(2.64,63.32)_ = 9.11, *p* < 0.01, η^2^ = 0.28, indicating that each group showed different eye-movement responses depending on the stimulus type. Further analysis revealed that the innocent-aware group showed a significant difference in dwell time among all stimulus types, *F*_(1.12,15.69)_ = 8.56, *p* < 0.05, η^2^ = 0.38. Specifically, the innocent-aware group spent more time gazing at crime-relevant and crime irrelevant stimuli than neutral stimuli, *t*_(14)_ = 2.80, *p* < 0.05, *t*_(14)_ = 3.23, *p* < 0.05 (Figure 2). On the other hand, the guilty and innocent-unaware groups showed similar eye-movement regardless of stimulus type, *F*_(2,32)_ = 1.41, *n.s*., *F*_(2,36)_ = 2.48, *n.s*. In addition, we conducted analyses to compare dwell time on each stimulus type among the three groups for exploratory analysis. As a result, we found that dwell time was a significantly more for crime-relevant stimuli,t for the innocent-aware and innocent-unaware groups than for the guilty group, *t*_(30)_ = 2.41, *p* < 0.05, *t*_(34)_ = 2.20, *p* < 0.05. In addition, the innocent-aware group spent more time gazing at crime-irrelevant stimuli than the guilty group, *t*_(30)_ = 2.46, *p* < 0.05, and spent less time gazing at neutral stimuli than the innocent-unaware group, *t*_(24)_ = 2.50, *p* < 0.05.

There was a significant main effect for the stimuli, *F*_(1.32,63.32)_ = 9.06, *p* < 0.01, η^2^ = 0.14, indicating that there was a statistically significant difference among stimuli in dwell time. Further analysis revealed that there was no difference between crime-relevant and crime-irrelevant stimuli (*n.s*.), while the dwell time for both crime-relevant and the crime-irrelevant stimuli were significantly longer than that for the neutral stimuli (*P* < 0.05 for both stimuli). There was no significant main effect for the group, *F*_(2,48)_ = 1.28, *n.s*.

### First Fixation Time

**Figure 3:**
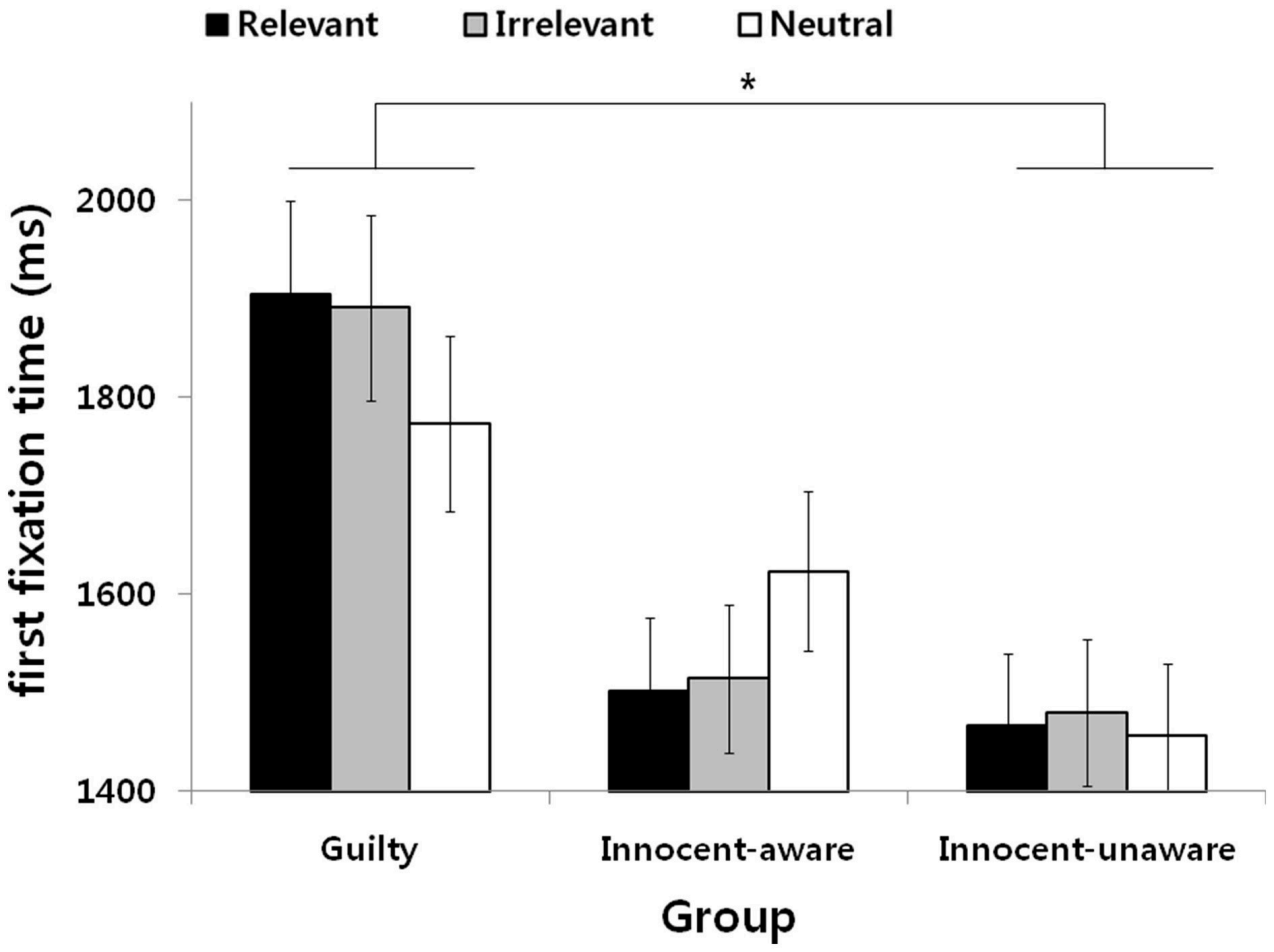
First fixation time for the three stimulus types with respect to the subject group. Means and standard error (^*^p < 0.05) are shown.

The results revealed no significant group × stimuli interaction, *F*_(4,96)_ = 1.23, *n.s*., and no main effect for stimuli, *F*_(2,96)_ = 0.02, *n.s*. However, there was a significant main effect for the group, *F*_(2,48)_ = 3.57, *p* < 0.05, η^2^ = 0.13. A LSD *post-hoc* test revealed that the first fixation time for the guilty group was significantly longer than that for the innocent-unaware group (*p* < 0.05) and marginally longer than that for the innocent-aware group (*p* = 0.06), indicating that the guilty group showed an avoidance tendency from all stimulus types, unlike the other groups (Figure 3).

## Discussion

The purpose of the present study was to investigate the attentional bias for guilty knowledge in the guilty group in the GKT using an eye tracker. In addition, the study aimed to examine whether eye-movement measurement can compensate for the defect of the GKT, in which innocent subjects who are exposed to guilty knowledge, may be judged as guilty.

The main finding of the present study is that the guilty group showed avoidance responses from all stimuli in a lie detection situation. Thus, our first hypothesis that the guilty group would show a shorter first fixation time and a shorter dwell time toward crime-relevant stimuli than crime-irrelevant and neutral stimuli, was rejected. In the present study, the guilty group showed no differences in dwell time for all stimulus types. They spent less time gazing at crime-relevant stimuli than the innocent-aware and innocent-unaware groups and spent less time gazing at crime-irrelevant stimuli than the innocent-aware group. Although there were no differences in dwell time, the guilty group spent less time gazing at all types of stimuli than the other two groups, indicating that they showed attentional avoidance from all stimuli. This finding is partially consistent with that of a previous study showing that guilty knowledge demands attention (24). In their experiments, Verschuere et al. (24) found that probe responses were slower in guilty knowledge trials than in neutral trials in a probe classification task, indicating general interruption of attentional performance in guilty knowledge trials, but no spatial shifting of attention. They concluded that it remains possible that participants may shift their attention away from guilty knowledge to try to avoid detection, and this result may support this prediction. We might assume that the guilty group did not involve all presented stimuli because of fear of regarding the deception detection situation *per se*. This lack of involvement is in agreement with a hesitation response during deception, which is one of the cognitively demanding tasks, such as gaze aversion (33), fewer body movements (34), and long pauses in statements between the lie detector's questions and responses.

The second important finding is that the innocent-aware group showed attentional bias toward crime-relevant and crime-irrelevant stimuli. Thus, our second hypothesis that the innocent-aware group would show a shorter first fixation time and a longer dwell time toward both crime-relevant and crime-irrelevant stimuli than neutral stimuli was partially supported. In the present study, they focused their attention on crime-relevant stimuli and crime-irrelevant stimuli longer than neutral ones. In addition, they observed crime-relevant stimuli for a longer time than the guilty group and observed neutral stimuli for less time than the innocent-unaware group. In the case of crime-relevant stimuli, such stimuli were significant to both the guilty and innocent-aware groups, but the guilty group avoided crime-relevant stimuli, unlike the innocent-aware group. This difference between the guilty and innocent-aware groups could possibly be interpreted to show the existence of the feeling of threat. The high level of threat for the crime-relevant stimuli in the guilty group under a lie detection situation may have contributed to the avoidance response, which is consistent with a previous study showing that the guilty group avoided guilty knowledge (30). In contrast, we may assume that those who had knowledge of crime information but did not commit the crime did not show an avoidance response toward crime-relevant stimuli because of the low level of threat under the lie detection test. Therefore, we assume that this indicates that mere knowledge plays an important role in allocating more attentional resources toward crime-relevant and crime-irrelevant stimuli in innocent-aware examinees, whereas actual action may have contributed to the attentional process for guilty participants.

Finally, the innocent-unaware group showed no differences in dwell time among all types of stimuli. Thus, our third hypothesis that there would be no differences in a first fixation time and a dwell time to all types of stimuli in the innocent-unaware group was partially supported. This is consistent with our prediction that there were no specific responses toward crime-relevant stimuli in the innocent-unaware group. Unlike the guilty group, the innocent-unaware group had not taken part in actual criminal action; thus, the presented stimuli might not have threatened them at all. In addition, the innocent-unaware group had no knowledge of criminal information, so there were no stimuli with significance or meaningfulness to them which might cause a threat or OR.

The present study has some implications. The guilty group, when faced with a lie detection situation showed a different pattern of attention from the innocent aware and the innocent-unaware group. This indicates that it is important to consider deception detection particularly with respect to nonverbal behaviors. Therefore, we should be careful when using detection of deception with visual stimuli since this avoidance response might cause problems, such as cheating the lie detection. In addition, attentional-avoidance patterns using eye-trackers can be used as an additional marker to distinguish deception from truth in criminal investigative settings.

The present study also has some limitations. First, it is difficult to generalize these findings to other populations and applied settings. This is because the study was conducted with undergraduate students in a mock-crime paradigm and addressed only one kind of mock-crime paradigm. Therefore, future research should be conducted with criminal suspects in a real deception detection setting and include more kinds of crimes. Second, we did not accurately and concretely assess physiological responses according to the stimulus type. Although we have controlled the valence and arousal of each stimulus type from a preliminary study, participants of the current study did not rate valence and arousal rate during this specific eye tracking experiment. Therefore, we cannot be sure that there were no differences between the stimulus types or between the groups. Future studies should rate the stimuli and measure physiological responses such as skin conductance responses and pupil sizes. Finally, although it constitutes a normal distribution, since the small sample size may elicit low statistical power, greater sample sizes may be useful in future research.

Despite these limitations, our findings may make up for the shortcomings of the GKT and provide important information on the effectiveness of the GKT as a lie detection technique. Namely, this study suggests that even if the innocent group is exposed to guilty knowledge, eye-tracking technology seems to be an effective method for distinguishing between deceptive groups and non-deceptive groups.

## Ethics Statement

The experiment was approved by the institutional review board in Chung-Ang University. All of the participants signed an informed consent that had been approved by the institutional review board in Chung-Ang University.

## Author Contributions

KK, GK, and J-HL conceived the experiment. KK and GK designed the experimental task and data acquisition of subjects. KK data analysis. KK, GK, and J-HL data interpretation. KK, GK, and J-HL drafting of the manuscript. All the authors revised the manuscript critically and provided final approval of the version to be published.

### Conflict of Interest Statement

The authors declare that the research was conducted in the absence of any commercial or financial relationships that could be construed as a potential conflict of interest.

## References

[1] Lykken DT Psychology and the lie detection industry. Am Psychol. (1974) 29:725–39. 10.1037/h0037441 4451301

[2] Elaad E. Effects of context and state of guilt on the detection of concealed crime information. Int J Psychophysiol. (2009) 71:225–34. 10.1016/j.ijpsycho.2008.10.001 18948149

[3] Verschuere B Ben-Shakhar G Meijer E Memory detection: theory and application of the concealed information test. In: Verschuere B, Ben-Shakhar G, editors. Theory of the Concealed Information Test. Cambridge, UK: Cambridge University Press (2011), p. 128–48.

[4] Lykken DT A Tremor in the Blood: Uses and Abuses of the Lie Detector. New York, NY: Plenum Trade (1998).

[5] Sokolov EN Perception and the Conditioned Reflex. New York, NY: MacMillan (1963).

[6] Furedy JJ. The concealed information test as an instrument of applied differential psychophysiology: methodological considerations. Appl Psychophysiol Biofeedback. (2009) 34:149–60. 10.1007/s10484-009-9097-y 19626435

[7] Ben-Shakhar G. The roles of stimulus novelty and significance in determining the electrodermal orienting response: interactive versus additive approaches. Psychophysiology. (1994) 31:402–11. 10.1111/j.1469-8986.1994.tb02448.x 10690920

[8] Ben-Shakhar G Elaad E. The validity of psychophysiological detection of information with the guilty knowledge test: a meta-analytic review. J Appl Psychol. (2003) 88:131–51. 10.1037/0021-9010.88.1.131 12675401

[9] Rosenfeld JP Hu X Labkovsky E Meixner J Winograd MR. Review of recent studies and issues regarding the P300-based complex trial protocol for detection of concealed information. Int J Psychophysiol. (2013) 90:118–34. 10.1016/j.ijpsycho.2013.08.012 24012907

[10] Meijer EH klein Selle N Elber L Ben-Shakhar G. Memory detection with the concealed information test: a meta analysis of skin conductance, respiration, heart rate, and P300 data. Psychophysiology. (2014) 51:879–904. 10.1111/psyp.12239 24916920

[11] Peth J Suchotzki K Gamer M. Influence of countermeasures on the validity of the Concealed Information Test. Psychophysiology. (2016) 53:1429–40. 10.1111/psyp.12690 27338719

[12] Ogawa T Matsuda I Tsuneoka M. The comparison question test versus the concealed information test? that was the question in japan: a comment on palmatier and rovner (2015). Int J Psychophysiol. (2015) 95:29–30. 10.1016/j.ijpsycho.2014.09.006 25242502

[13] Zaitsu W. External validity of concealed information test experiment: comparison of respiration, skin conductance, and heart rate between experimental and field card tests. Psychophysiology. (2016) 53:1100–7. 10.1111/psyp.12650 27031043

[14] Gamer M Gödert HW Keth A Rill H-G Vossel G Electrodermal and phasic heart rate responses in the Guilty Action Test: comparing guilty examinees to informed and uninformed innocents. Int J Psychophysiol. (2008) 69:61–8. 10.1016/j.ijpsycho.2008.03.001 18433904

[15] Zvi L Nachson I Elaad E. Effects of coping and cooperative instructions on guilty and informed innocents' physiological responses to concealed information. Int J Psychophysiol. (2012) 84:140–8. 10.1016/j.ijpsycho.2012.01.022 22330977

[16] Zvi L Nachson I Elaad E. Effects of perceived efficacy and prospect of success on detection in the Guilty Actions Test. Int J Psychophysiol. (2015) 95:35–45. 10.1016/j.ijpsycho.2014.12.010 25543067

[17] Selle NK Verschuere B Kindt M Meijer E Ben-Shakhar G Orienting versus inhibition in the concealed information test: different cognitive processes drive different physiological measures. Psychophysiology. (2015) 53:579–90. 10.1111/psyp.12583 26615984

[18] Ben-Shakhar G Gronau N Elaad E Leakage of relevant information to innocent examines in the GKT: an attempt to reduce false-positive outcomes by introducing target stimuli. J Appl Psychol. (1999) 84:651–60. 10.1037/0021-9010.84.5.651

[19] Ambach W Stark R Vaitl D. An interfering n-back task facilitates the detection of concealed information with EDA but impedes it with cardiopulmonary physiology. Int J Psychophysiol. (2011) 80:217–26. 10.1016/j.ijpsycho.2011.03.010 21440579

[20] Gamer M. Does the Guilty Actions Test allow for differentiating guilty participants from informed innocents? A re-examination. Int J Psychophysiol. (2010) 76:19–24. 10.1016/j.ijpsycho.2010.01.009 20114064

[21] Ryan JD Hannula DE, Cohen NJ. The obligatory effects of memory on eye movements. Memory. (2007) 15:508–25. 10.1080/09658210701391022 17613794

[22] Siddle DA. Orienting, habituation, and resource allocation: an associative analysis. Psychophysiology. (1991) 28:245–59. 10.1111/j.1469-8986.1991.tb02190.x 1946891

[23] Verschuere B Crombez G Degrootte T Rosseel Y Detecting concealed information with reaction times: validity and comparison with the polygraph. Appl Cogn Psychol. (2010) 24:991–1002. 10.1002/acp.1601

[24] Verschuere B Crombez G Koster EHW. Orienting to guilty knowledge. Cogn Emotion. (2004) 18:265–79. 10.1080/02699930341000095 29148303

[25] Hermans D Vansteenwegen D Eelen P Eye movement registration as a continuous index of attention deployment: data from a group of spider anxious students. Cogn Emotion. (1999) 13:419–34. 10.1080/026999399379249

[26] Schwedes C Wentura D. The revealing glance: eye gaze behavior to concealed information. Memory Cogn. (2012) 40:642–51. 10.3758/s13421-011-0173-1 22194248

[27] In-Albon T Kossowsky J Schneider S. Vigilance and avoidance of threat in the eye movements of children with separation anxiety disorder. J Abnormal Child Psychol. (2010) 38:225–35. 10.1007/s10802-009-9359-4 19813086

[28] Armstrong T Olatunji BO. Eye tracking of attention in the affective disorders: a meta-analytic review and synthesis. Clin Psychol. Rev. (2012) 32:704–23. 10.1016/j.cpr.2012.09.004 23059623 PMC3556338

[29] Mogg K Bradley BP Time course of attentional bias for fear-related pictures in spider-fearful individuals. Behav Res Ther. (2006) 44:1241–50. 10.1016/j.brat.2006.05.003 16870133

[30] Kim K Kim J Lee JH Guilt, lying, and attentional avoidance of concealed information. Soc Behav Personal. (2016) 44:1467–76. 10.2224/sbp.2016.44.9.1467

[31] Hartwig M Granhag PA Strömwall LA Vrij A. Detecting deception via strategic disclosure of evidence. Law Hum Behav. (2005) 29:469–84. 10.1007/s10979-005-5521-x 16133950

[32] Nicholls MER Orr CA Okubo M Loftus A Satisfaction guaranteed: the effects of spatial biases on responses to Likert scales. Psychol Sci. (2006) 17:1027–8. 10.1111/j.1467-9280.2006.01822.x 17201782

[33] Ekman P Telling Lies: Clues to Deceit in The Marketplace, Politics and Marriage. New York, NY: Norton and Company (2001).

[34] Ekman P Friesen WV Hand movements. J Commun. (1972) 22:353–74.

